# Association between Different Types of Edible Oils and Anthropometric Indices Mood, and Appetite among Women

**DOI:** 10.1155/2022/1233748

**Published:** 2022-10-19

**Authors:** Nahid Kangani, Mohammadreza Mohammadi, Mobina Zeinolabedin, Nick Bellissimo, Leila Azadbakht

**Affiliations:** ^1^Department of Community Nutrition, School of Nutritional Sciences and Dietetics, Tehran University of Medical Sciences (TUMS), Tehran, Iran; ^2^Toronto Metropolitan University, School of Nutrition, Toronto M5B-2K3, Canada

## Abstract

**Introduction:**

The objective of this study was to evaluate the relationship between consumption of dietary oils and anthropometric indices, mood, and appetite among women staff of Tehran University of Medical Sciences.

**Methods:**

A cross-sectional study design was used, and 245 women staff of Tehran University of Medical Sciences participated. A 168-item food frequency questionnaire was used to evaluate dietary and nutrient intake. The association between liquid vegetable oils, hydrogenated vegetable oil, and animal fat intake and anthropometric indices, appetite, and mood was evaluated. The Profile of Mood States (POMS) questionnaire was used to assess mood. A Visual Analogue Scale (VAS) was used to evaluate appetite status. The tape measure was used to measure the waist circumference and height. SPSS was used to compute body mass index (BMI) and waist-to-height ratio (WHtR).

**Results:**

In the present study, sunflower and frying oil were the most consumed liquid oils (*n* = 135/245 participants). Participants with a moderate intake of MUFA had greater odds ratio (OR: 3.47; 95% CI: 1.20–10.7; *P* trend = 0.025) of a high appetite compared to those with a low intake of MUFA. However, the study found no evidence of an association between consumption of edible oils (vegetable oils, animal fat oils, and other fatty acid sources) and mood, anthropometric indices, or appetite.

**Conclusions:**

In the current research, we noticed a significant connection between moderate intake of MUFA and a large appetite and no association between consumption of edible oils and other outcomes. In conclusion, a balanced diet low in fast meals, processed foods, cakes, cookies, and sweets is suggested to limit the consumption of artificial trans-fatty acids.

## 1. Introduction

The prevalence of overweight and obesity is a major health concern worldwide, which continues to gradually increase [1]. In 2016, the World Health Organization (WHO) reported that over 1.9 billion people were overweight and over 650 million people were obese [2]. A study published in 2020, based on data from STEPs 2016, found that the incidence of obesity and overweight/obesity in Iranian adults was reported to be 22.7% and 59.3%, respectively, and the prevalence of obesity was higher among women compared to men [3].

Based on global health trends and the prevalence of chronic and noncommunicable diseases, managing and preventing obesity is critical for public health [4]. Due to the nature of their occupation, sedentary lifestyle [5], and likely dietary changes, employees have expressed worry about their general health and anthropometric indices [6]. In particular, women with obesity in Asia were subjected to harsh public criticism, severe social pressure, and discrimination based on their weight and appearance [7, 8]. Some factors that influence obesity risk include heredity, physical activity, stress and mood, dietary habits, and lifestyle changes [9].

Poor morale and mood can impair social interaction, work quality, and appetite [10]. Increasing fruit and vegetable consumption, whole grains, legumes, nuts, polyunsaturated fatty acids (PUFA), and omega-3 fatty acids and decreasing simple carbs, fast meals, and saturated fatty acids have been found to have a favorable effect on mood and sadness [11]. Two RCTs studies have examined the influence of medium-chain triglycerides (MCT) and conjugated linoleic acid (CLA) on overweight and fullness, which shows consumption of MCT reduces energy intake in the subsequent 48 h, whereas CLA does not [12]. The other one has shown that data on the appetite effects of CLA is limited but does suggest potential [13].

Among macronutrients, fats have the highest energy density. Due to observed correlations between excessive fat intake and obesity, there has been increased attention on understanding the association between consumption of various types of fatty acids and obesity or fat storage [14]. In the population, liquid vegetable oils (such as olive oil, canola oil, corn oil, sunflower oil, sesame oil, etc.), solid vegetable oils, and animal fat are commonly consumed. Research has suggested that the consumption of omega-3 fatty acids improves mood and depression [15]. A study also found that a high omega-6:omega-3 PUFA ratio was associated with excessive fat mass and unfavorable metabolic indices [14]. Monounsaturated fatty acids (MUFA) have been shown to lower abdominal obesity compared to PUFA, which is essential for treating and preventing metabolic syndrome [16]. However, consumption of PUFA may improve appetite regulation [17]. Commonly used oils, such as olive oil, have been found to help support the maintenance of body weight [18, 19]. In addition, canola and sunflower oils have been shown to effectively lower total cholesterol, LDL, and triglyceride levels and increase HDL while having no effect on body weight [19]. Conversely, hydrogenated vegetable oils have been found to raise the risk of metabolic syndrome [20].

To the best of our knowledge, previous work has not evaluated the relationship between consumption of hydrogenated oils and mood, anthropometrics, and appetite. Therefore, we hypothesized that dietary habits and consuming healthy fats will have a positive effect on improving anthropometrics, poor mood, and high appetite. The purpose of this research was to investigate the relationship between the intake of edible oils and anthropometric indices, mood, and hunger in female staff members of Tehran University of Medical Sciences.

## 2. Materials and Methods

### 2.1. Study Design and Subjects

A cross-sectional study design was used and included female staff members (*n* = 245) from the Tehran University of Medical Sciences. Simple random sampling was used to select the participants. To determine the sample size appropriate for this investigation, we considered the prevalence of obesity among Iranian adults as the primary dependent variable [21]. The required sample size was calculated by first retrieving data on the prevalence of obesity from previous studies and then using the following formula:

(1) $$\begin{array}{c} N=\frac{\left[{\left(z1-\alpha /2\right)}^{2}\times \left(p1\left(1-p1\right)\right)\right]}{d^{2}},\\ N=\frac{\left[{\left(1.96\right)}^{2}\times \left(17.5\times 82.5\right)\right]}{25}=221. \end{array}$$

With *d* = 5, the number of samples with alpha = 0.05 and *p*=17.5 percent was computed. Due to the likelihood of under- and over-reporting of subjects, 245 participants were included in our study.

The inclusion criteria were as follows: willingness to participate (all participants provided online informed consent); those between 30 and 50 years of age; absence of chronic diseases including diabetes, hyperlipidemia, thyroid disease, cardiovascular disease, kidney disease, malignancy, hypertension, and acute liver disease; and no use of corticosteroids, thyroid medications, neuropsychiatric medications, metformin, or allergy medications. Furthermore, participants were not pregnant, lactating, or in menopause and were not eligible if they had reported following a special diet in the three months prior. Finally, participants were excluded from the study during analysis if their self-reported energy intake was <800 kcal/day or >4,200 kcal/day. All data were self-reported and collected using an online questionnaire. The Human Ethical Committee of Tehran University of Medical Science approved the study protocol (IR.TUMS.MEDICINE.REC.1400.241).

### 2.2. Data Collection Tools

Due to the COVID-19 pandemic, we were unable to gather in-person data. Therefore, all data were collected online through widely used, reliable, and validated questionnaires. The data collection online link had seven sections: (1) the consent form, (2) anthropometric characteristics, (3) assessment of socioeconomic status, (4) physical activity questionnaire (IPAQ) [22], (5) appetite questionnaire (VAS) [23], (6) mood assessment questionnaire (POMS) [24], and (7) food frequency questionnaire (FFQ) [25].

### 2.3. Dietary Intake

We measured edible oils intake including vegetable oils and animal oils using FFQ-168 food items to estimate routine consumption of dietary oils by participants (Table 1). Detailed instructions on accurately reporting the quantity and type of oil consumed were provided to the participant prior to initiating the questionnaire. Participants were asked how frequently they consumed each food item on the FFQ over the past year. To improve simplicity and efficiency, participants were provided with nine options for frequency (three options in a day, three options in a week, and three options in a month). The Nutritionist IV (N4) nutrient analysis software was utilized to determine the total calories and nutrients consumed by each participant. Finally, total daily grams, total energy intake, and nutritional counts were entered into SPSS for statistical analysis. Finally, the following food confounders were extracted from the FFQ questionnaire: B vitamins, iron, and zinc (that increase appetite); calcium, caffeine, and fiber (that reduce obesity, WHtR, and waist circumference); and iron, B vitamins, magnesium, and zinc (that support mood).

### 2.4. Demographic and Socioeconomic Status

For this purpose, the socioeconomic status and demographic questionnairewas applied, which had questions on marital status, education, job, family size, means of support, and method of transportation. Each questionnaire item was coded in order to calculate the socioeconomic status score, and the codes were added. The score was divided into three groups, and people were placed into one of three categories depending on their socioeconomic status: poor, middle class, or rich.

### 2.5. Anthropometric Indices

Height (cm), weight (kg), and waist circumference (cm) were measured by the participants, and detailed instructions were provided. To measure body weight, the participants were instructed to stand in the center of the scale while wearing minimal clothing and be barefoot. To measure height, the participants were instructed to use a height meter while barefoot, place the soles of their feet on the ground with their heels pressed together, place their hand next to their torso with their shoulders relaxed, and place their spine, buttocks, and the back of their heels in contact with the height gauge or wall. To measure waist circumference, participants were instructed to wear no clothing around their abdomen or as little clothing as possible. Participants were instructed to ensure that the tape measure was parallel to the ground and to identify the waist prior to measuring, which was classified as the smallest portion of the trunk, typically from the navel or above. SPSS was used to compute body mass index (BMI) and waist-to-height ratio (WHtR). Finally, the classification of general and abdominal obesity followed the WHO recommended cut-offs [26].

### 2.6. Appetite

A Visual Analogue Scale (VAS) questionnaire was used to evaluate subjective appetite during past 6 months. This questionnaire included four questions that measured desire for food, hunger, fullness, and prospective food consumption. The individuals select a number from two options, not hungry at all (0 points) and have not been so hungry (100 points). To facilitate response, we investigated five options (Table 2). The overall score was calculated by taking the sum of all of the scores and dividing by 4; therefore, it varied from 0 to 100. We were unable to identify a usual cut-off for hunger upon a review of the literature; therefore, we categorized the scores into tertiles for analysis. A score >55 was classified as a voracious appetite (third tertile); a score between 45 and 55 was classified as a moderate appetite (second tertile); and a score <45 was classified as a poor appetite (first tertile).

### 2.7. Mood and Emotions

The POMS questionnaire was utilized to evaluate mood throughout the preceding year. This questionnaire includes 65 items organized into 6 categories: anxiety, depression, tiredness, confusion, anger, and ability. This questionnaire's Likert scale ranged from 0 (absolutely not) to 4 (very high). To obtain an overall mood score, the scores of the 5 negative mood categories (anxiety, depression, anger, fatigue, and confusion) were added together, and the score of the positive mood category (ability) was subtracted. The overall mood score ranged from −22 to 177, with a lower score suggesting a more positive mood. Since we were also unable to identify a usual cut-off for mood, mood scores were classified into tertiles as follows: a score >38 indicates a poor mood (third tertile), a score between 11 and 38 indicates a middling mood (second tertile), and a score <11 indicates a great mood.

### 2.8. Physical Activity

To assess physical activity level, we used the short-form International Physical Activity Questionnaire (IPAQ). IPAQ consisted of seven questions. Overall, the questions evaluated the number of days and minutes of participation in light and heavy activities and the average time spent walking and sitting over the past seven days. Physical activity level <600 METs-min/week was classified as low physical activity; that between 600 and 3,000 METs-min/week was classified as moderate physical activity; and that >3,000 METs-min/week was classified as high physical activity.

### 2.9. Statistical Analysis

The FFQ data were analyzed by the Nutritionist IV software, and we obtained the amount of consumed oils and fatty acids (MUFA, PUFA, omega-3, omega-6, linoleic acid, olive oil, vegetable oils, animal fat, etc.). There were no other separate groups of vegetable oils (liquid and solid) to explore the link between the factors and these oils. Tertiles were conducted on total vegetable liquid oil, animal fat, fatty acids, and trans-fatty acid sources.

Two tables were achieved from variables analysis in fatty acid (*w*3—it was obtained from linolenic acid, EPA, and DHA—MUFA, and *w*3/*w*6 ratio) and trans-fatty acid sources tertiles (natural—from animal sources, artificial, and total sources). Scores of the variables in these tertiles are shown in Tables 3 and 4 (based on covariance analysis). Also, the odds ratio and 95% CI of variables are shown in Tables 5 and 6 (based on logistic regression). In these tables, omega-3 is totalized from linolenic acid, EPA, and DHA.

SPSS software (22.0; SPSS Inc.) was used for all statistical data analysis. A histogram and the Kolmogorov–Smirnov test were used to determine the normality of the variable distribution. A one-way ANOVA was used to evaluate the association between the consumption of various edible oils as independent variables (i.e., vegetable liquid oil, hydrogenated vegetable oil, and animal fat) and anthropometric indices, mood, and appetite as dependent variables. For descriptive characteristics (i.e., supplement and drug use, socioeconomic level, etc.), the chi-square test was used to compare how individuals were distributed throughout the groups. An analysis of covariance was also used to compensate for the confounding influence of physical activity. The Bonferroni correction was used if the differences were significant. Linear and nonlinear regression models were used to assess the relationship between dietary oil consumption and anthropometric indicators, hunger, and mood. We used a binary logistic regression model to estimate the association between edible oil consumption in two categories, vegetable oil and animal fat oil, and outcomes including poor mood, high appetite, waist circumference, overweight and obesity, and waist-to-height ratio. An odds ratio (OR) with a 95% confidence interval (CI) was reported in three statistical models: crude, model 1, and model 2, which were specific to the outcome variable.

High appetite was adjusted for energy intake, age, infected with COVID-19 in the past, and body mass index in model 1 and adjusted for model 1 + micronutrients (thiamine, niacin, vitamin B12, iron, zinc, fiber, and simple sugar), supplements (zinc, multivitamin, iron + vitamin D, iron, iron + multivitamin, zinc + vitamin D, and multivitamin + zinc + omega-3), socioeconomic status, and macronutrient (fiber + simple sugar) in model 2. The poor mood was adjusted for energy, age, infected with COVID-19, body mass index, and socioeconomic status in model 1 and adjusted for model 1 + micronutrients (thiamine, riboflavin, niacin, B6, folate, vitamin B12, iron, zinc and magnesium, fiber, and simple sugar) and supplements (vitamin D, zinc, multivitamin, iron + vitamin D, iron, iron + multivitamin, zinc + vitamin D, multivitamin + calcium, and multivitamin + zinc + omega-3), and macronutrient (fiber + simple sugar) in model 2. Overweight and obesity were adjusted for energy, age, infected with COVID-19, and socioeconomic status in model 1 and adjusted for model 1 + micronutrients (calcium, fiber, caffeine, and simple sugar), supplements (vitamin D, zinc, multivitamin, calcium, iron + vitamin D, iron, iron + multivitamin, zinc + vitamin D, multivitamin + calcium-D, multivitamin + zinc + omega-3, and iron + calcium), and macronutrient (fiber + simple sugar) in model 2. Finally, waist circumference and waist-to-height ratio were adjusted for energy, age, infected with COVID-19, body mass index, and socioeconomic status in model 1 and adjusted for micronutrients (calcium and caffeine), supplements (vitamin D, zinc, multivitamin, calcium, iron + vitamin D, iron, iron + multivitamin, zinc + vitamin D, multivitamin + calcium-D, multivitamin + zinc + omega-3, and iron + calcium), and macronutrient (fiber + simple sugar) in model 2.

## 3. Results

### 3.1. Participant Characteristics

The participants' mean age was 38.7 ± 6.4 years, and their mean BMI, waist circumference, and waist-to-height ratio were 24.8 ± 3.9 kg/m^2^, 80 ± 13 cm, and 0.49 ± 0.07 cm, respectively. Due to this study being conducted during the COVID-19 outbreak, the incidence of COVID-19 in participants was assessed from 2020 to 2021. Eighty-three participants (33.9%) were infected with COVID-19 during the previous year, and the history of infection was adjusted as a confounder in our models. Participants reported mostly low levels of physical activity (54.7%), with only 4% classified as having high physical activity. A total of 44.5% of participants (*n* = 109) were in the low socioeconomic status group.  Table 7 represents the sociodemographic characteristics of the participants.

### 3.2. Dietary Intake of Participants

The average consumption of energy, macronutrients, fatty acids, vitamins, minerals, and dietary groups across the three tertiles of edible oils consumption are presented in Table 8. All of these (excluding energy intake) are adjusted for energy intake and reported as mean ± SD. The average daily energy consumption and daily fat intake were 2,039.7 ± 614.1 kcal/d and 70.8 ± 25.8 g/d, respectively. Intake of fat and monosaturated fatty acids was significant in both group of animal fat and vegetable liquid oil consumption (*P* value < 0.001), while intake of protein and carbohydrate was just significant in the group of vegetable oil consumption (*P* value < 0.001).

### 3.3. Consumption of Liquid Vegetable Oils

Sunflower and frying oils were the most often consumed liquid oils in this study (*n* = 135 reporting consumption). Sesame oil was also typical (*n* = 25 reporting consumption), while three respondents did not report using any liquid vegetable oils. Olive oil use was separated from other liquid oils in the FFQ. We observed that while many participants (*n* = 167) utilized olive oil, the average daily consumption was low (2.8 ± 3.5 g/d). The consumption of liquid vegetable oils is presented in Table 1.

### 3.4. Appetite across Tertiles of Dietary Oils and Fatty Acids and Trans-Fatty Acid Sources

Tables 3, 4, and 9 provide evidence that the mean score of appetite was insignificant across tertiles of edible oils consumption and different fatty acid source. In the final model, the insignificant association was also observed in vegetable oils groups (OR: 2.50; 95% CI: 1.02–36.11; *P* trend = 0.712) and in animal fat oils groups (OR: 1.98; 95% CI: 0.88–5.55; *P* trend = 0.141; Table 10). Participants with a moderate intake of MUFA were four times more likely (OR: 4.10; 95% CI: 1.31–12.7; *P* trend = 0.022) to have a high appetite compared to those with low consumption of MUFA. However, in all other groups, the association between highest intake of *w*3 (OR: 1.10; 95% CI: 0.44–2.74; *P* trend = 0.899) and *w*3/*w*6 ratio (OR: 0.62; 96% CI: 0.28–1.42; *P* trend = 0.124) were insignificant compared to those without consumption in final models (Tables 5 and 6). Furthermore, the association between the highest consumption of manufactured fatty acids (OR: 0.87; 95% CI: 0.33–2.26; *P* trend = 0.724) or natural fatty acids (OR: 1.23; 95% CI: 0.49–3.09; *P* trend = 0.843) and a high appetite were insignificant (Tables 5 and 6).

### 3.5. Mood across Tertiles of Dietary Oils and Fatty Acids and Trans-Fatty Acid Sources

The mean mood score was not significantly different among tertiles of edible oil and fatty acid intake, except for the group of participants who consumed MUFA (*P* value = 0.053; Tables 3, 4, and 9). There were no significant associations between the consumption of liquid vegetable oils (OR: 1.4; 95% CI: 0.6–3.4; *P* trend = 0.726) or animal oils (OR: 0.5; 95% CI: 0.2–1.7; *P* trend = 0.787) and poor mood when compared to participants who reported low levels of consumption (i.e., tertile 1; Table 10). Similar associations were observed for associations between other various fatty acid sources and poor mood including *w*3 (OR: 1.13; 95% CI: 0.46–2.75; *P* trend = 0.827), MUFA (OR: 2.47; 95% CI: 0.96–6.33; *P* trend = 0.365), and *w*3/*w*6 (OR: 1.13; 95% CI: 0.62–2.87; *P* trend: 0.199). Finally, there was no evidence of a significant association between high consumption of natural fatty acids (OR: 0.41; 95% CI: 0.14–1.17; *P* trend = 0.199) or artificial fatty acids sources (OR: 0.74; 95% CI: 0.30–1.82; *P* trend = 0.318) and poor mood (Tables 5 and 6).

### 3.6. Anthropometric Indices across Tertiles of Edible Oils and Different Fatty Acids Sources

There was no evidence of a significant association between dietary oil consumption and anthropometric indices across tertiles of intake, with the exception of those who consumed artificial fatty acids sources (*P* value = 0.016; Tables 7 and 9).

There was no significant association between the use of large quantities of liquid oil and anthropometric indices including overweight and obesity (OR: 1.03; 95% CI: 0.49–2.1; *P* trend = 0.989), WC (OR: 1.47; 95% CI: 0.6–2.7; *P* trend = 0.626), or WTHR (OR: 1.29; 95% CI: 0.6–2.7; *P* trend = 0.628) compared to those that consumed low amounts of liquid oil (i.e., tertile 1). Similar associations were observed for the association between animal oil consumption and overweight and obesity (OR: 0.88; 95% CI: 0.4–1.7; *P* trend = 0.826), WC (OR: 0.46; 95% CI: 0.3–1.26; *P* trend = 0.280), and WHtR (OR: 0.46; 95% CI: 0.3–1.28; *P* trend = 0.280; Table 10).

Various fatty acids sources including *w*3 (OR: 0.75; 95% CI: 0.35–1.60; *P* trend = 646), MUFA (OR: 0.87; 95% CI: 0.39–1.92; *P* trend = 0.952), *w*3/*w*6 ratio (OR: 0.68; 95% CI: 0.34–1.36; *P* trend = 0.275), natural fatty acids (OR: 1.10; 95% CI: 0.48–2.55; *P* trend = 0.466), and artificial fatty acids source (OR: 0.91; 95% CI: 0.42–1.91; *P* trend = 0.143) were not significantly associated with overweight and obesity (Tables 5 and 6).

There was no evidence of a significant association between consuming various fatty acid sources including *w*3 (OR: 1.49; 95% CI: 0.63–3.54; *P* trend = 0.111), MUFA (OR: 1.01; 95% CI: 0.38–2.64; *P* trend = 0.694), *w*3/*w*6 ratio (OR: 0.77; 95% CI: 0.34–1.7; *P* trend = 0.490), natural fatty acids (OR: 1.41; 95% CI: 0.53–3.72; *P* trend = 0.792), or artificial fatty acids (OR: 0.89; 95% CI: 0.38–2.18; *P* trend = 0.634), and odds of a high WC (Tables 5 and 6).

Similarly, there was no evidence of a significant association between consumption of *w*3 (OR: 0.92; 95% CI: 0.43–1.99; *P* trend = 0.910), MUFA (OR: 0.82; 95% CI: 0.36–1.84; *P* trend = 0.623), *w*3/*w*6 ratio (OR: 1.07; 95% CI: 0.54–2.11; *P* trend = 0.377), natural fatty acids sources (OR: 1.32; 95% CI: 1.32–0.57; *P* trend = 0.643), and artificial fatty acids sources (OR: 0.84; 95% CI: 0.39–1.83; *P* trend = 0.505), and odds of having a high waist-to-height ratio (Tables 5 and 6).

## 4. Discussion

To the best of our knowledge, this is the first study that has investigated the association between edible oils and anthropometric indices, mood, and appetite in the female staff of Tehran University of Medical Sciences. The present study found no significant associations between the consumption of edible oils or fatty acid sources and mood, anthropometric indices, or appetite. However, individuals who consumed moderate amounts of MUFA were more likely to have a higher appetite.

Dietary oils and fats are important sources of energy and different types of fatty acids are necessary for physiological functions. In contrast, overconsumption of dietary oils may have adverse effects on health, such as an increased risk of chronic diseases, diabetes, and obesity [27]. In the present study, energy intake was higher with increased consumption of vegetable liquid oils and animal fat. Natural fats and oils are a combination of MUFA, PUFA, and saturated fatty acids [28]. The results of this study indicated that the intake of PUFA increased with the consumption of liquid vegetable oils. A study assay to determine the fatty acid composition of several vegetable oils found that oils, such as corn oil (PUFA: 48 ± 4.5 g/d) or sunflower oil (PUFA: 59.5 ± 7.5 g/d), were considered to have a high amount of PUFA [29]. Furthermore, our findings suggested that MUFA and saturated fatty acids intake was higher with higher consumption of animal fats. Previous work by Gilani et al. evaluating animal oils [30] and Nazari et al. [31] found similar results (SFA: 56 ± 4.1 g/d, MUFA: 24.8 ± 1.8 g/d).

There are many studies that have examined the relationship between olive oil consumption and chronic disease risk [32–34]. In the present study, 167 participants reported consuming olive oil, but the amount of olive oil consumed by participants (2.88 ± 3.5 g/d) was much lower than the average intake of olive oil (22.7 ± 44 g/d) reported in a previous literature review of studies that examined the effects of the Mediterranean diet on health and diseases [35].

Appetite is an important factor to be considered for the regulation of body weight. Many factors such as medications, mood, dietary choices, neurotransmitters, and hormones such as leptin, ghrelin, glucagon-like peptide (GLP-1), cholecystokinin (CCK), and YY peptide (PYY) are involved in controlling appetite [36]. Findings in the present study suggested that higher consumption of MUFA from various dietary sources increased the risk of having a high appetite. Our findings are consistent with a comprehensive review of the literature that found that MUFA consumption was linked to a weaker PYY response compared to PUFA consumption and suggested that a high MUFA diet may enhance appetite [37]. However, other research evaluating the role of fatty acid consumption on appetite has contradicted our findings. A study that evaluated 12 overweight patients with type 2 diabetes found that MUFA (i.e., the predominant fatty acid in olive oil) had a greater effect on GLP-1 stimulation when compared to saturated fatty acid (i.e., found in butter) [38]. GLP-1 is one of the primary hormones that influences and regulates satiety. In other words, the findings suggested that consuming MUFA would result in greater GLP-1 stimulation, theoretically resulting in a lower appetite compared to consuming saturated fatty acids. Another study found that the reduction in ghrelin (hunger hormone) after PUFA and MUFA consumption was significantly greater when compared to saturated fatty acid consumption [39]. However, a clinical trial found that consuming high amounts of PUFA, MUFA, or trans-fatty acids had no effect on appetite or energy consumption in overweight subjects [40]. The conflicting findings may be due to differences in study design, populations, and measurement tools. For instance, the clinical experiment was conducted over a brief period of time (3 days), or perhaps overweight persons were less impacted by manipulation and dietary modifications in the lab setting with calorie meter. A study evaluating 40 normal-weight adults found that PUFA consumption resulted in stronger appetite control compared to consuming MUFA or saturated fatty acids [38]. One of the possible mechanistic reasons for the inverse relationship between PUFA consumption and body weight or appetite may be the association with fatty acid beta-oxidation changes [38, 41] and increased mitochondrial respiration of liver cells and cardiac and skeletal muscle [42]. Finally, a study by Kozimor et al. with an RCT design observed an increase in satiety after consuming saturated fatty acid sources compared to MUFA [43]. However, the conflicting findings from this study may be due to the very high percentage of fat used in this study (providing 70% of a person's energy from fat) compared to other studies.

The present study found no relationship between dietary oil or fatty acid consumption and mood. Many studies have found that the Mediterranean diet, which is low in trans- and saturated-fatty acids and rich in omega-3 fatty acids, was associated with better mood and lower levels of depression [32, 44, 45]. Some studies have found that the Western diet, characterized as high consumption of red meat, processed foods, fast foods, and sweets, and low consumption of fruits and vegetables, was associated with poor mental health [46–48]. In the present study, participants may be more aware of their consumption of processed foods compared to the general population resulting in more controlled consumption. Previous work has found a reduced risk of depression and mental disorders with omega-3 fatty acids intake [49–51]. Unexpectedly, our findings did not suggest that omega-3 fatty acid intake was associated with mood. However, it is important to consider that this relationship has been found in randomized controlled trials and clinical trials evaluating omega-3 supplementation, whereas observational studies have suggested conflicting findings [49–51].

According to our findings, obesity was related to the higher intake of artificial sources of trans-fatty acids. Furthermore, in our study, a higher intake of omega-3 fatty acids was associated with decreased odds of having abdominal adiposity (i.e., high waist circumference). Various studies have shown that dietary fat intake is positively associated with the risk of having overweight or obesity [52, 53]. However, the consumption of calories from fat alone may only have a small effect on weight, and rather, the type of fatty acid, especially trans-fatty acid, saturated-fatty acid, and animal fats, is a more important factor [54]. A study aiming to determine the relationship between different types of fat intake and long-term weight changes in 121,000 American adults found that higher intakes of saturated- and trans-fatty acids were directly associated with weight gain in both men and women [28]. Furthermore, another study observed that high consumption of artificial trans-fatty acids prevented weight loss, especially in women [55], which is aligned with findings in previous studies and the present study. Other studies have also shown that the consumption of artificial trans-fatty acids was associated with obesity in children [56, 57]. Trans-fatty acids have been suggested to increase body mass index and waist circumference by altering some SNPs (single nucleotide polymorphisms) of the FTO (fat mass and obesity-associated) gene [58]. These fatty acids have also been associated with changes in intestinal microbiota [59], which may influence obesity risk.

A study by Micallef et al. found that plasma omega-3 fatty acids were inversely associated with BMI, waist circumference, and hip circumference, especially in obese individuals [60]. Furthermore, a study by Haghravan et al. in 50 overweight women found that body weight, body fat percentage, and waist circumference were significantly reduced in those that received omega-3 supplementation compared to the control group [61]. A possible mechanistic explanation for these findings may be increased fat oxidation [62] and increased satiety after omega-3 intake [63]. However, other studies with RCT design have found no association between omega-3 intake and changes in weight and waist circumference [64, 65].

The present study has limitations that are important to consider in the interpretation of the findings. The study design was cross-sectional, which does not allow for the determination of cause-and-effect relationships between dietary oils or fatty acid consumption and appetite, mood, and anthropometric indices; also, our low sample size of the study led to a decrease in the study power and the lack of significance of the main results. To collect dietary intake from participants, an FFQ was used, which is subject to recall bias, social desirability bias, and a possibility of over- or under-estimating dietary intake. However, we mitigated the risk of over- and under-reporting by establishing reasonable energy intake cut-offs prior to statistical analysis. The study population consisted of female staff of Tehran University of Medical Sciences in Iran, and thus, the findings are not generalizable to males or other populations. Notably, many questions within the questionnaires used in this study may be subject to errors or changes in response due to participant burden. To address this possibility, the answers of each person were checked by a research assistant, and in the case of a concern, we called the person to verify the answers. Finally, while we collected important demographic information, we did not account for all the possible factors (e.g., smoking status) that may have influenced the associations.

The strengths of the present study were that it was, to the best of our knowledge, the first study to evaluate the relationship between dietary oils consumption and anthropometric indices, mood, and appetite in women staff of Tehran University of Medical Sciences. The food frequency questionnaire used in this study had 168 items and is validated, reliable, and covered most of the foods commonly consumed by participants. Also, in this study, physical activity state and the relationship between fatty acids (omega-3, MUFA, and omega-3/omega-6 ratios) and trans-fatty acid sources (natural, artificial, and total sources) were surveyed by appetite, mood, and anthropometric indices. Finally, our analyses were adjusted for many important and clinically relevant confounders (such as nutrients, supplements, energy, etc.).

## 5. Conclusion

In the present study, we found a significant association between moderate intake of MUFA and a higher appetite but no evidence of an association between dietary oils consumption and anthropometric indices or mood. A balanced diet low in fast food meals, processed foods, cakes, cookies, and sweets is suggested to limit the consumption of artificial trans-fatty acids.

## Figures and Tables

**Table 1:** Intake of vegetable liquid oils in participants (g/d).

Type of edible oil	Number of consumers	Mean ± SD in total sample	Mean ± SD in consumers
Sunflower oil	52	1.74 (4.2)	8.2 (5.7)
Canola oil	7	0.18 (1.2)	6.4 (3.9)
Frying oil	39	1.25 (3.6)	7.8 (5.7)
Corn oil	6	0.16 (1.2)	6.7 (4.3)
Cooking oil	18	0.39 (1.8)	5.3 (4.8)
Sesame oil	25	0.79 (3)	7.7 (5.9)
Rice bran oil	1	0.003 (0.05)	0.8
Sunflower + frying oil	44	1.38 (3.7)	7.8 (5.3)
Sunflower + sesame oil	7	0.18 (1.1)	6.4 (1.2)
Sunflower + cooking oil	3	0.06 (0.5)	5 (1.7)
Frying + cooking oil	25	0.82 (3)	8 (5.7)
Frying + sesame oil	13	0.42 (2.2)	7.9 (6.3)
Frying + canola oil	2	0.04 (0.4)	5.3
No consumed	3	—	—

Olive oil	167	1.96 (3.2)	2.8 (3.5)

**Table 2:** Visual analogue scale for the appetite questionnaire.

How hungry do you feel?	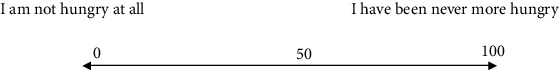
How satisfied do you feel?	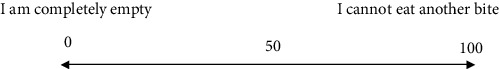
How full do you feel?	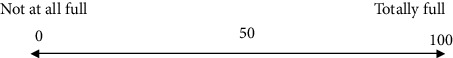
How much do you think you can eat?	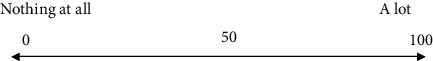
How much food do you intend to consume?	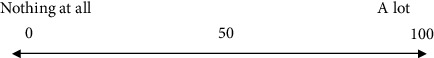

**Table 3:** Appetite, mood, and anthropometric indices in tertiles of different fatty acids.

			Tertiles of different fatty acids											
Variable			Tertile of *w*3 (g/d)			*P* value	Tertile of MUFA (g/d)			*P* value	Tertile of *w*3/*w*6 ratio (g/d)			*P* value
		Participants	*T*1: <0.34	*T*2: 0.34–0.49	*T*3: >0.49		*T*1: <18.4	*T*2: 18.4–24.2	*T*3: >24.2		*T*1: <0.03	*T*1:0.03–0.04	*T*3: >0.04	
		*N* = 245	*N* = 81	*N* = 82	*N* = 82		*N* = 81	*N* = 82	*N* = 82		*N* = 75	*N* = 86	*N* = 84	
Appetite score	Crude	51.8 (13.2)	51.3 (14.4)	53.5 (10.5)	50.7 (14.5)	0.378	50.6 (12.7)	52.5 (12.5)	52.4 (14.4)	0.601	52.1 (13.8)	53.7 (11.9)	49.6 (13.8)	0.124
	^1^Model 1	—	51.6 (1.5)	53.1 (1.4)	50.7 (1.5)	0.510	50 (1.6)	52.4 (1.4)	53 (1.6)	0.446	52.1 (1.4)	53.5 (1.3)	49.9 (1.3)	0.182
	^2^Model 2	—	50.9 (1.52)	53.1 (1.44)	51.5 (1.62)	0.544	49.6 (1.68)	52.6 (1.43)	53.3 (1.8)	0.318	52.2 (1.49)	53.5 (1.39)	49.7 (1.39)	0.158

Mood score	Crude	29.5 (32.7)	21.9 (29.7)	29 (30.6)	37.6 (35.7)	0.008	25.2 (28.2)	23.6 (30.7)	39.7 (36.3)	0.002	29.3 (36.5)	26.3 (28.2)	33 (33.3)	0.403
	^3^Model 1	—	25.5 (3.7)	30.4 (3.5)	32.6 (3.8)	0.420	32.1 (4)	24.4 (3.5)	32.1 (4.1)	0.210	29.2 (3.6)	27 (3.4)	32.4 (3.4)	0.534
	^4^Model 2	—	28.01 (3.5)	30.67 (3.35)	30.0 (3.81)	0.853	28.98 (3.91)	23.2 (3.32)	36.50 (4.18)	0.053	30.21 (3.50)	27.35 (3.23)	31.2 (3.27)	0.689

Overweight and obesity	Crude	24.8 (3.9)	24.7 (3.7)	24.9 (3.5)	24.6 (4.5)	0.905	24.8 (3.8)	24.7 (3.7)	24.8 (4.3)	0.996	25 (4.2)	24.7 (3.7)	24.6 (3.9)	0.803
	^5^Model 1	—	24.7 (0.4)	25 (0.4)	24.6 (0.4)	0.781	24.7 (0.4)	24.7 (0.4)	24.9 (0.5)	0.948	24.9 (0.4)	24.8 (0.4)	24.5 (0.4)	0.797
	^6^Model 2	—	24.8 (0.45)	24.9 (0.43)	24.6 (0.48)	0.923	24.5 (0.49)	24.5 (0.42)	25.3 (0.52)	0.548	25.3 (0.44)	24.8 (0.41)	24.2 (0.41)	0.224

Waist circumference	Crude	80 (13)	79 (13.3)	80.3 (12.3)	80.6 (13.5)	0.700	80.4 (14.7)	80.4 (10.9)	79.2 (13.3)	0.792	80 (12.7)	80.2 (14.1)	79.8 (12.3)	0.977
	^3^Model 1	—	78.6 (1.2)	79.9 (1.1)	81.4 (1.2)	0.332	80.7 (1.3)	80.4 (1.1)	78.9 (1.3)	0.669	79.4 (1.2)	80.4 (1.1)	80.1 (1.1)	0.823
	^6^Model 2	—	78.5 (1.54)	80.0 (1.47)	81.5 (1.64)	0.468	80.3 (1.71)	79.8 (1.71)	79.9 (1.80)	0.978	80.9 (1.53)	80.2 (1.43)	78.9 (1.421)	0.626

Waist-to-height ratio	Crude	0.49 (0.07)	0.48 (0.08)	0.49 (0.07)	0.49 (0.08)	0.724	0.49 (0.08)	0.49 (0.06)	0.48 (0.10)	0.579	0.49 (0.07)	0.49 (0.08)	0.49 (0.07)	0.935
	^3^Model 1	—	0.4 (0.009)	0.4 (0.009)	0.4 (0.009)	0.417	0.4 (0.010)	0.4 (0.009)	0.4 (0.010)	0.811	0.4 (0.009)	0.4 (0.008)	0.4 (0.008)	0.774
	^6^Model 2	—	0.48 (0.009)	0.49 (0.009)	0.50 (0.10)	0.460	0.49 (0.010)	0.49 (0.009)	0.48 (0.011)	0.932	0.49 (0.009)	0.49 (0.008)	0.48 (0.009)	0.633

^*∗*^The crude model was resulted from one-way ANOVA, and the numbers are reported as mean ± SD. ^*∗*^Models 1 and 2 were resulted from covariance analysis, and the numbers are reported as mean ± SE. ^*∗*^Model 2 is the main model. *P*^*∗*^*P* value <0.05 shows a significant level of association. ^1^Adjusted for energy, age, infected with COVID-19, and body mass index; ^2^model 1 + micronutrients (thiamine, niacin, vitamin B12, iron, zinc, and magnesium), supplements (zinc, multivitamin, iron + vitamin D, iron, iron + multivitamin, zinc + vitamin D, and multivitamin + zinc + omega-3), and macronutrient (fiber and simple sugar); ^3^adjusted for energy, age, infected with COVID-19, body mass index, and socioeconomic status; ^4^number 3 + micronutrients (thiamine, riboflavin, niacin, B6, folate, vitamin B12, iron, and zinc), supplements (vitamin D, zinc, multivitamin, iron + vitamin D, iron, iron + multivitamin, zinc + vitamin D, multivitamin + calcium-D, and multivitamin + zinc + omega-3), and macronutrient (fiber and simple sugar); ^5^adjusted for energy, age, infected with COVID-19, and socioeconomic status; and ^6^number 5 + micronutrients (calcium and caffeine), supplements (vitamin D, zinc, multivitamin, calcium, iron + vitamin D, iron, iron + multivitamin, zinc + vitamin D, multivitamin + calcium-D, multivitamin + zinc + omega-3, and iron + calcium), and macronutrient (fiber and simple sugar).

**Table 4:** Appetite, mood, and anthropometric indices in tertiles of different sources of trans-fatty acids.

		Tertiles of different sources of trans-fatty acids												
Variable			Tertile of natural source (g/d)			*P* value	Tertile of artificial source (g/d)			*P* value	Tertile of total source (g/d)			*P* value
		Participants	*T*1: <341.4	*T*2: 341.4–482.2	*T*3: >482.2		*T*1: <39.8	*T*2: 39.8–71.4	*T*3: >71.4		*T*1: <402.1	*T*1:402.1–549.7	*T*3: >549.7	
		*N* = 245	*N* = 81	*N* = 82	*N* = 82		*N* = 81	*N* = 82	*N* = 82		*N* = 81	*N* = 82	*N* = 82	
Appetite score	Crude	51.8 (13.2)	53.3 (14.5)	50.7 (12.5)	51.4 (12.7)	0.421	51.4 (11.7)	52.1 (13.4)	51.9 (14.6)	0.939	52.6 (13.8)	50.9 (12.9)	52 (13.1)	0.710
	^1^Model 1	—	53.3 (1.4)	51.2 (1.4)	51 (1.4)	0.497	52.5 (1.5)	51.3 (1.4)	51.7 (1.5)	0.843	52.6 (1.5)	51.1 (1.4)	51.8 (1.5)	0.770
	^2^Model 2	—	54.3 (1.56)	51.1 (1.43)	50 (1.62)	0.174	52.3 (1.54)	51.9 (1.45)	51.3 (1.62)	0.909	52.8 (1.57)	51.3 (1.44)	51.3 (1.66)	0.733

Mood score	Crude	29.5 (32.6)	32.2 (33.1)	32.5 (33.8)	23.9 (30.7)	0.165	20.8 (31.2)	29.3 (32)	38.3 (32.6)	0.003	28.9 (31.1)	30.6 (34.4)	29 (32.7)	0.930
	^3^Model 1	—	37.6 (3.5)	34.1 (3.4)	17 (3.5)	<0.0001	25.2 (3.7)	30.5 (3.5)	32.8 (3.8)	0.384	35 (3.6)	33.5 (3.4)	20.1 (3.7)	0.015
	^4^Model 2	—	33.6 (3.97)	30.6 (3.39)	24.5 (4.26)	0.399	28.4 (3.62)	31.4 (3.3)	29.2 (3.85)	0.854	29.3 (3.99)	30.9 (3.36)	28.4 (4.2)	0.889

Overweight and obesity	Crude	24.8 (3.9)	24.9 (4.3)	24.1 (3.2)	25.2 (4.1)	0.174	24 (3.3)	25.5 (3.8)	24.8 (4.4)	0.050	24.8 (4.2)	24.5 (3.4)	25 (4.2)	0.685
	^5^Model 1	—	24.9 (0.4)	24.1 (0.4)	25.3 (0.4)	0.169	23.7 (0.4)	25.5 (0.4)	25.1 (0.4)	0.012	24.7 (0.4)	24.4 (0.4)	25.1 (0.4)	0.625
	^6^Model 2	—	25.3 (0.5)	24.3 (0.42)	24.7 (0.53)	0.268	23.9 (0.45)	25.6 (0.41)	24.8 (0.48)	0.016	25.3 (0.5)	24.4 (0.42)	24.6 (0.53)	0.330

Waist circumference	Crude	80 (13)	78.9 (15.8)	78.9 (10.5)	82.1 (12)	0.186	79.5 (10.1)	80.9 (15.2)	79.6 (13.3)	0.763	78.7 (15.5)	80.5 (9.6)	80.8 (13.3)	0.546
	^3^Model 1	—	78.3 (1.2)	80 (1.1)	81.6 (1.2)	0.198	81 (1.2)	79.4 (1.1)	79.5 (1.2)	0.618	78.3 (1.2)	80.9 (1.1)	80.7 (1.2)	0.252
	^6^Model 2	—	80 (1.75)	78.9 (1.47)	81.1 (1.86)	0.639	79 (1.59)	80.9 (1.45)	80 (1.68)	0.649	80.1 (1.74)	80 (1.48)	79.9 (1.86)	0.998

Waist-to-height ratio	Crude	0.49 (0.07)	0.48 (0.09)	0.49 (0.06)	0.50 (0.07)	0.237	0.48 (0.06)	0.49 (0.09)	0.48 (0.07)	0.667	0.48 (0.09)	0.49 (0.05)	0.49 (0.07)	0.582
	^3^Model 1	—	0.4 (0.009)	0.4 (0.009)	0.5 (0.009)	0.107	0.4 (0.009)	0.4 (0.009)	0.4 (0.009)	0.518	0.4 (0.009)	0.4 (0.009)	0.5 (0.009)	0.340
	^6^Model 2	—	0.49 (0.011)	0.48 (0.009)	0.5 (0.011)	0.586	0.48 (0.010)	0.49 (0.009)	0.49 (0.010)	0.580	0.49 (0.010)	0.49 (0.009)	0.49 (0.011)	0.991

^*∗*^The crude model was resulted from one-way ANOVA, and the numbers are reported as mean ± SD. ^*∗*^Models 1 and 2 was resulted from covariance analysis, and the numbers are reported as mean ± SE. ^*∗*^Model 2 is the main model. *P*^*∗*^*P* value <0.05 shows a significant level of association. ^1^Adjusted for energy, age, infected with COVID-19, and body mass index; ^2^model 1 + micronutrients (thiamine, niacin, vitamin B12, iron, zinc, and magnesium), supplements (zinc, multivitamin, iron + vitamin D, iron, iron + multivitamin, zinc + vitamin D, and multivitamin + zinc + omega-3), and macronutrient (fiber and simple sugar); ^3^adjusted for energy, age, infected with COVID-19, body mass index, and socioeconomic status; ^4^number 3 + micronutrients (thiamine, riboflavin, niacin, B6, folate, vitamin B12, iron, and zinc), supplements (vitamin D, zinc, multivitamin, iron + vitamin D, iron, iron + multivitamin, zinc + vitamin D, multivitamin + calcium-D, and multivitamin + zinc + omega-3), and macronutrient (fiber and simple sugar); ^5^adjusted for energy, age, infected with COVID-19, and socioeconomic status; and ^6^number 5 + micronutrients (calcium and caffeine), supplements (vitamin D, zinc, multivitamin, calcium, iron + vitamin D, iron, iron + multivitamin, zinc + vitamin D, multivitamin + calcium-D, multivitamin + zinc + omega-3, and iron + calcium), and macronutrient (fiber and simple sugar).

**Table 5:** Odds ratio and 95% confidence interval for unfavorable variables in tertiles of different fatty acids.

		Tertiles of different fatty acids											
Variable		Tertile of *w*3 (g/d)			*P* trend	Tertile of MUFA (g/d)			*P* trend	Tertile of *w*3/*w*6 ratio (g/d)			*P* trend
		*T*1: <0.34	*T*2: 0.34–0.49	*T*3: >0.49		*T*1: <18.4	*T*2: 18.4–24.2	*T*3: >24.2		*T*1: <0.03	*T*2:0.03–0.04	*T*3: >0.04	
		*N* = 81	*N* = 82	*N* = 82		*N* = 81	*N* = 82	*N* = 82		*N* = 75	*N* = 86	*N* = 84	
High appetite	Crude	1	1.2 (0.6–2.3)	1.2 (0.1–2.5)	0.643	1	0.9 (0.4–1.9)	0.6 (0.3–1.6)	0.254	1	1 (0.5–2.1)	1.6 (0.8–3.3)	0.133
	^1^Model 1	1	1.2 (0.6–2.5)	1.2 (0.5–2.7)	0.581	1	0.7 (0.3–1.6)	0.3 (0.1–1)	0.054	1	1.1 (0.5–2.1)	1.6 (0.8–3.3)	0.175
	^2^Model 2	1	1.18 (0.4–3.2)	1.10 (0.4–2.7)	0.899	1	4.10 (1.3–12.7)	2.38 (0.8–6.4)	0.022	1	0.65 (0.27–1.52)	0.62 (0.28–1.4)	0.124

Poor mood	Crude	1	0.7 (0.3–1.5)	0.3 (0.2–0.7)	0.006	1	1 (0.4–2)	0.3 (0.1–0.7)	0.003	1	1.1 (0.5–2.2)	0.7 (0.3–1.4)	0.383
	^3^Model 1	1	0.9 (0.4–1.8)	0.6 (0.3–1.4)	0.318	1	1.3 (0.6–3)	0.8 (0.3–2.1)	0.752	1	1.1 (0.5–2.3)	0.7 (0.3–1.5)	0.449
	^4^Model 2	1	1.11 (0.43–2.85)	1.13 (0.46–2.75)	0.826	1	1.91 (0.63–5.80)	2.47 (0.96–6.33)	0.305	1	1.12 (0.49–2.57)	1.13 (0.62–2.88)	0.739

Overweight and obesity	Crude	1	0.9 (0.4–1.7)	1 (0.5–1.9)	0.946	1	0.8 (0.4–1.6)	1 (0.5–2)	0.821	1	1 (0.5–1.9)	1.1 (0.6–2.1)	0.651
	^5^Model 1	1	0.8 (0.4–1.7)	1 (0.5–2.2)	0.860	1	0.8 (0.4–1.7)	1 (0.4–2.5)	0.881	1	0.9 (0.5–1.8)	1.1 (0.6–2.2)	0.606
	^6^Model 2	1	0.81 (0.46–1.79)	0.75 (0.35–1.60)	0.646	1	0.95 (0.38–2.39)	0.87 (0.39–1.92)	0.952	1	0.68 (0.33–1.37)	0.68 (0.34–1.36)	0.275

High waist circumference	Crude	1	0.6 (0.3–1.4)	0.4 (0.2–1)	0.066	1	1 (0.5–2.3)	0.9 (0.4–1.9)	0.883	1	1 (0.4–2.1)	1 (0.5–2.2)	0.851
	^3^Model 1	1	0.5 (0.1–1.6)	0.2 (0.08–0.9)	0.047	1	1.1 (0.4–3.4)	1.4 (0.3–5.8)	0.565	1	0.7 (0.2–2)	0.8 (0.2–2.3)	0.710
	^6^Model 2	1	2.15 (0.83–5.53)	1.49 (0.63–3.54)	0.111	1	0.81 (0.27–2.45)	1.01 (0.38–2.64)	0.694	1	0.75 (0.32–1.72)	0.77 (0.34–1.71)	0.490

Waist-to-height ratio	Crude	1	0.9 (0.4–1.7)	0.9 (0.4–1.7)	0.804	1	0.9 (0.5–1.8)	1.1 (0.6–2.2)	0.578	1	1.4 (0.7–2.6)	1 (0.5–1.9)	0.886
	^3^Model 1	1	0.8 (0.4–1.6)	0.8 (0.4–1.8)	0.680	1	0.9 (0.4–1.8)	1.1 (0.5–2.7)	0.730	1	1.3 (0.6–2.6)	1 (0.5–2)	0.871
	^6^Model 2	1	1.03 (0.46–2.29)	0.92 (0.43–1.99)	0.910	1	0.78 (0.31–1.98)	0.82 (0.36–1.84)	0.623	1	0.72 (0.35–1.45)	1.07 (0.54–2.11)	0.377

^*∗*^The *P* trend was reported from logistic regression, and the results are based on the odds ratio or OR (95% CI). ^*∗*^Model 2 is the main model. *P*^*∗*^*P* value <0.05 shows a significant level of association. ^1^Adjusted for energy, age, infected with COVID-19, and body mass index; ^2^model 1 + micronutrients (thiamine, niacin, vitamin B12, iron, and zinc) + macronutrient (fiber and simple sugar), supplements (zinc, multivitamin, iron + vitamin D, iron, iron + multivitamin, zinc + vitamin D, multivitamin + zinc + omega-3) and socioeconomic status; ^3^adjusted for energy, age, infected with COVID-19, body mass index, and socioeconomic status; ^4^number 3 + micronutrients (thiamine, riboflavin, niacin, B6, folate, vitamin B12, iron, zinc, and magnesium), and macronutrient (fiber and simple sugar), supplements (vitamin D, zinc, multivitamin, iron + vitamin D, iron, iron + multivitamin, zinc + vitamin D, multivitamin + calcium-D, and multivitamin + zinc + omega-3); ^5^adjusted for energy, age, infected with COVID-19, and socioeconomic status; and ^6^number 5 + micronutrients (calcium and caffeine), macronutrient (fiber and simple sugar), supplements (vitamin D, zinc, multivitamin, calcium, iron + vitamin D, iron, iron + multivitamin, zinc + vitamin D, multivitamin + calcium-D, multivitamin + zinc + omega-3, and iron + calcium).

**Table 6:** Odds ratio and 95% confidence interval for unfavorable variables in tertiles of trans-fatty acids.

		Tertiles of different sources of trans-fatty acids											
Variable		Tertile of natural sources (g/d)			*P* trend	Tertile of artificial sources (g/d)			*P* trend	Tertile of total sources (g/d)			*P* trend
		*T*1: <341.4	*T*2: 341.4–482.2	*T*3: >482.2		*T*1: <39.8	*T*2: 39.8–71.4	*T*3: >71.4		*T*1: <402.1	*T*1: 402.1–549.7	*T*3: >549.7	
		*N* = 81	*N* = 82	*N* = 82		*N* = 81	*N* = 82	*N* = 82		*N* = 81	*N* = 86	*N* = 82	
High appetite	Crude	1	1.5 (0.7–3)	1.1 (0.5–1.9)	0.959	1	0.6 (0.3–1.2)	0.8 (0.4–1.6)	0.645	1	1.5 (0.7–3.1)	0.8 (0.4–1.6)	0.645
	^1^Model 1	1	1.3 (0.6–2.8)	0.9 (0.4–1.9)	0.921	1	0.7 (0.3–1.5)	0.8 (0.3–2)	0.766	1	1.4 (0.7–3)	0.7 (0.3–1.5)	0.446
	^2^Model 2	1	0.91 (0.33–2.5)	1.23 (0.49–3.09)	0.843	1	1.35 (0.48–3.80)	0.87 (0.33–2.26)	0.724	1	1.73 (0.61–5.60)	2.16 (0.83–5.60)	0.391

Poor mood	Crude	1	0.8 (0.4–1.5)	1.2 (0.6–2.5)	0.468	1	0.6 (0.3–1.2)	0.4 (0.2–0.7)	0.009	1	0.8 (0.4–1.6)	0.8 (0.4–1.6)	0.652
	^3^Model 1	1	0.9 (0.4–1.9)	3.2 (1.4–7.5)	0.007	1	0.7 (0.3–1.5)	0.8 (0.3–1.8)	0.650	1	0.9 (0.4–1.9)	2.3 (1–5.3)	0.060
	^4^Model 2	1	0.39 (0.11–1.35)	0.41 (0.14–1.17)	0.199	1	0.87 (0.33–2.30)	0.74 (0.30–1.82)	0.825	1	0.74 (0.23–2.39)	0.63 (0.23–1.68)	0.717

Overweight and obesity	Crude	1	1.4 (0.7–2.7)	0.8 (0.4–1.6)	0.683	1	0.6 (0.3–1.1)	0.7 (0.3–1.3)	0.298	1	1.1 (0.6–2.2)	0.9 (0.4–1.7)	0.803
	^5^Model 1	1	1.4 (0.7–2.8)	0.8 (0.4–1.6)	0.654	1	0.5 (0.2–1)	0.5 (0.2–1.1)	0.093	1	1.1 (0.6–2.2)	0.8 (0.4–1.7)	0.711
	^6^Model 2	1	0.67 (0.24–1.80)	1.10 (0.48–2.55)	0.466	1	1.80 (0.76–4.29)	0.91 (0.42–1.91)	0.143	1	0.60 (0.22–1.63)	0.93 (0.4–2.14)	0.283

High waist circumference	Crude	1	1.2 (0.5–2.8)	0.6 (0.3–1.4)	0.276	1	0.7 (0.3–1.6)	0.7 (0.3–1.4)	0.367	1	0.8 (0.4–1.8)	0.6 (0.2–1.3)	0.202
	^3^Model 1	1	0.6 (0.2–1.9)	0.5 (0.1–1.8)	0.353	1	1.7 (0.5–4.9)	1.4 (0.4–4.8)	0.492	1	0.4 (0.1–1.4)	0.4 (0.1–1.6)	0.214
	^6^Model 2	1	0.90 (0.30–2.68)	1.41 (0.53–3.72)	0.792	1	1.24 (0.45–3.42)	0.89 (0.36–2.18)	0.634	1	1 (0.33–3.05)	1.18 (0.46–3.05)	0.976

Waist-to-height ratio	Crude	1	1.3 (0.7–2.5)	0.8 (0.4–1.5)	0.573	1	0.7 (0.4–1.4)	0.8 (0.4–1.5)	0.573	1	1.1 (0.6–2.2)	0.9 (0.5–1.8)	0.929
	^3^Model 1	1	1.3 (0.7–2.6)	0.7 (0.3–1.5)	0.481	1	0.6 (0.3–1.2)	0.6 (0.2–1.3)	0.195	1	1.1 (0.6–2.2)	0.8 (0.4–1.8)	0.751
	^6^Model 2	1	0.84 (0.31–2.25)	1.32 (0.57–3.03)	0.643	1	1.28 (0.54–3.06)	0.84 (0.39–1.83)	0.505	1	0.59 (0.23–1.59)	0.91 (0.39–2.11)	0.267

^*∗*^The *P* trend was reported from logistic regression, and the results are based on the odds ratio or OR (95% CI). ^*∗*^Model 2 is the main model. *P*, ^*∗*^*P* value <0.05 shows a significant level of association. ^1^Adjusted for energy, age, infected with COVID-19, and body mass index; ^2^model 1 + micronutrients (thiamine, niacin, vitamin B12, iron, and zinc), macronutrient (fiber and simple sugar), supplements (zinc, multivitamin, iron + vitamin D, iron, iron + multivitamin, zinc + vitamin D, and multivitamin + zinc + omega-3), and socioeconomic status; ^3^adjusted for energy, age, infected with COVID-19, body mass index, and socioeconomic status; ^4^number 3 + micronutrients (thiamine, riboflavin, niacin, B6, folate, vitamin B12, iron, zinc, and magnesium), macronutrient (fiber and simple sugar), supplements (vitamin D, zinc, multivitamin, iron + vitamin D, iron, iron + multivitamin, zinc + vitamin D, multivitamin + calcium-D, and multivitamin + zinc + omega-3); ^5^adjusted for energy, age, infected with COVID-19, and socioeconomic status; and ^6^number 5 + micronutrients (calcium and caffeine), macronutrient (fiber and simple sugar), supplements (vitamin D, zinc, multivitamin, calcium, iron + vitamin D, iron, iron + multivitamin, zinc + vitamin D, multivitamin + calcium-D, multivitamin + zinc + omega-3, and iron + calcium).

**Table 7:** Characteristics of participants in tertiles of vegetable liquid oil and animal fat.

		Tertiles of edible oils								
Variable			Tertile of vegetable liquid oil (g/d)			*P* value	Tertile of animal fat (g/d)			*P* value
		Participants	*T*1: <6	*T*2: 6–9	*T*3: >9		*T*1: <2.08	*T*2: 2.08–4.48	*T*3: >4.48	
		*N* = 245	*N* = 85	*N* = 81	*N* = 79		*N* = 59	*N* = 78	*N* = 108	
Quantitative variables/mean ± SD										
Age (year)		37.8 (6.4)	38.2 (6.6)	40.1 (6.2)	37.7 (6.1)	0.045	38.9 (6.7)	38.2 (6.4)	38.9 (6.1)	0.787
Weight (kg)		65.5 (11.5)	65.8 (11.6)	64.8 (10.9)	65.8 (12.2)	0.799	65.4 (11.4)	66.1 (12.4)	65.1 (11.1)	0.828
Height (cm)		162.4 (5.4)	161.8 (5.5)	162.1 (5)	163.2 (5.8)	0.205	163.2 (5.7)	162 (5.8)	162.2 (5)	0.393
Waist (cm)		80.0 (13.0)	78.9 (14.4)	80.8 (10.9)	80.4 (13.5)	0.630	78.8 (16.2)	81 (12.8)	79.9 (11.2)	0.611
BMI (kg/m^2^)		24.8 (3.9)	25.1 (3.9)	24.5 (3.6)	24.6 (4.2)	0.675	24.5 (3.9)	25.1 (4.1)	24.7 (3.7)	0.635
Waist-to-height ratio (cm)		0.49 (0.07)	0.48 (0.08)	0.49 (0.06)	0.49 (0.08)	0.705	0.48 (0.09)	0.50 (0.07)	0.49 (0.06)	0.473
Physical activity (MET-min/week)		929.7 (1,178.4)	1,017.4 (1,285.6)	776.5 (735.4)	992.4 (1,404.5)	0.358	1,078 (1,226.5)	971 (1,507.6)	818.6 (830.4)	0.370
Qualitative variable/*N* (%)										

Supplement	Yes	108 (44.1)	30 (27.8)	46 (42.6)	32 (29.6)	0.015	37 (33)	40 (43.6)	60 (60.4)	0.408
	No	137 (55.9)	55 (40.1)	35 (25.5)	47 (34.3)		22 (20.4)	38 (35.2)	48 (44.4)	

Physical activity	Low	134 (54.7)	46 (34.3)	44 (32.8)	44 (32.8)	0.591	24 (17.9)	44 (32.8)	66 (49.3)	0.071
	Medium	101 (41.2)	34 (33.7)	36 (35.6)	31 (30.7)		33 (32.7)	29 (28.7)	39 (38.6)	
	High	10 (4.1)	5 (50)	1 (10)	4 (40)		2 (20)	5 (50)	3 (30)	

Marital status	Yes	162 (66.1)	56 (34.6)	54 (33.3)	52 (32.1)	0.992	32 (39)	51 (51.6)	79 (71.4)	0.047
	No	83 (33.9)	29 (34.9)	27 (32.5)	27 (32.5)		27 (32.5)	27 (32.5)	29 (34.9)	

Socioeconomic status	Low	109 (44.5)	38 (34.9)	36 (33)	35 (32.1)	0.435	31 (28.4)	32 (29.4)	46 (42.2)	0.617
	Medium	67 (27.3)	27 (40.3)	17 (25.4)	23 (34.3)		12 (17.9)	24 (35.8)	31 (46.3)	
	High	69 (28.2)	20 (29)	28 (40.6)	21 (30.4)		16 (23.2)	22 (31.9)	31 (44.9)	

Education	High school	1 (0.4)	1 (100)	0	0	0.442	1 (100)	0	0	0.261
	Diploma	7 (2.9)	4 (57.1)	2 (28.6)	1 (14.3)		1 (14.3)	4 (57.1)	2 (28.6)	
	College	237 (96.7)	80 (33.8)	79 (33.3)	78 (32.9)		57 (24.1)	74 (31.2)	106 (44.7)	

Infected with COVID-19	Yes	83 (33.9)	25 (30.1)	29 (34.9)	29 (34.9)	0.556	20 (24.1)	29 (34.9)	34 (41)	0.720
	No	162 (66.1)	60 (37)	52 (32.1)	50 (30.9)		39 (24.1)	49 (30.2)	74 (45.7)	

^*∗*^The *P* value reported for the quantitative variables was resulted from one-way ANOVA, and the numbers are reported as mean ± SD. ^*∗*^The *P* value for the qualitative variables was calculated by the chi-square test, and the results are based on *N* (%).^*∗*^*PP* value <0.05 shows a significant level of association.

**Table 8:** Dietary intake of participants in tertiles of liquid vegetable oil and animal fat.

	Tertiles of edible oils							
Variable	Tertile of vegetable liquid oil (g/d)			*P* value	Tertile of animal fat (g/d)			*P* value
	*T*1: <6	*T*2: 6–9	*T*3: >9		*T*1: <2.08	*T*2: 2.08–4.48	*T*3: >4.48	
	*N* = 85	*N* = 81	*N* = 79		*N* = 59	*N* = 78	*N* = 108	
Energy (kcal/d)	1,853.7 (559.7)	1,950.1 (519.2)	2,331.6 (658.5)	<0.0001	1,956.9 (617.4)	1,936.2 (481.3)	2,159.6 (673.7)	0.024
Carbohydrate (g/d)	260.87 (81.43)	273.68 (77.26)	319.16 (114.59)	<0.0001	284.05 (111.27)	269.01 (77.42)	249.58 (96.73)	0.195
Protein (g/d)	70.59 (22.02)	73.38 (21.51)	86.07 (25.12)	<0.0001	74.32 (22.59)	75.93 (21.45)	78.12 (26)	0.596
Fat (g/d)	62.33 (25.08)	66.38 (21.86)	84.59 (25.40)	<0.0001	62.82 (23.76)	65.43 (18.93)	79.15 (28.93)	0.0001
Fiber (g/d)	15.32 (0.56)	17.37 (6.79)	20.95 (9.95)	<0.0001	18.56 (9.32)	16.09 (5.77)	18.66 (8.28)	0.065
Caffeine (mg/d)	106.79 (77.03)	99.9 (82.11)	127.96 (94.20)	0.094	102.60 (82.84)	111.45 (81.94)	111.04 (88.73)	0.623
Linoleic acid (g/d)	10.09 (5.05)	11.27 (4)	17.50 (6.77)	<0.0001	13.01 (7.83)	11.92 (4.86)	13.48 (6.16)	0.240
*α*-Linolenic acid (g/d)	0.320 (0.178)	0.370 (0.236)	0.501 (0.297)	<0.0001	0.356 (0.280)	0.372 (0.187)	0.433 (0.271)	0.103
*w*3/*w*6 ratio	0.040 (0.012)	0.039 (0.013)	0.035 (0.013)	0.017	0.034 (0.012)	0.039 (0.011)	0.040 (0.014)	0.025
SFA (g/d)	22.42 (9.46)	23.34 (9.10)	26.66 (9.65)	0.010	18.86 (7.05)	22.39 (6.84)	28.10 (10.67)	0.0001
MUFA (g/d)	20.36 (8.64)	21.87 (8.08)	27.47 (8.39)	<0.0001	20.50 (7.67)	21.18 (6.67)	26.03 (10.04)	0.0001
PUFA (g/d)	12.66 (5.60)	14.12 (4.59)	20.59 (7.52)	<0.0001	15.62 (8.63)	14.49 (5.22)	16.47 (6.88)	0.022
Cholesterol (mg/d)	272.47 (130.78)	278.78 (96.23)	330.40 (147.66)	0.007	258.01 (135.19)	285.74 (115.06)	317.90 (130.36)	0.013

Vitamins								
Thiamine (mg)	1.67 (0.45)	1.82 (0.51)	2.11 (0.82)	<0.0001	1.79 (0.612)	1.8 (0.580)	1.94 (0.648)	0.212
Riboflavin (mg)	1.99 (0.74)	2.09 (0.73)	2.47 (1.05)	<0.0001	1.99 (0.709)	2.20 (0.816)	2.63 (0.986)	0.166
Niacin (mg)	19.24 (5.93)	21.10 (7.73)	24.61 (10.03)	<0.0001	20.75 (7.52)	20.76 (6.90)	22.64 (9.49)	0.209
Vitamin B6 (mg)	1.52 (0.69)	1.65 (0.72)	2.05 (1.05)	<0.0001	1.73 (0.072)	1.59 (0.682)	1.84 (0.971)	0.143
Vitamin B9 (*μ*g)	294.45 (128.91)	325.48 (123.10)	403.88 (214.09)	<0.0001	331.02 (164.73)	317.59 (138.79)	361.09 (182.15)	0.188
Vitamin B12 (*μ*g)	4.20 (1.92)	4.51 (1.67)	6.62 (6.84)	<0.0001	4.15 (1.91)	5.44 (4.33)	5.34 (5.06)	0.153
Vitamin C (mg)	114.56 (67.98)	128.19 (68.75)	154.07 (93.15)	0.004	138.68 (88.10)	113.48 (56.66)	141.80 (58.06)	0.040

Minerals								
Calcium (mg)	893.29 (398.03)	893.37 (325.21)	1.011.61 (385.28)	<0.0001	870.58 (339.68)	973.63 (386.62)	934.27 (381.05)	0.279
Magnesium (mg)	238.60 (77.49)	248.84 (76.07)	300.95 (111.29)	<0.0001	261 (106.53)	250.32 (72.89)	271.18 (97.95)	0.320
Zinc (mg)	8.80 (3.05)	9.26 (3.05)	10.92 (3.51)	<0.0001	9.31 (3.10)	9.59 (2.99)	9.84 (3.66)	0.608
Fe (mg)	13.62 (4.09)	14.57 (4.43)	17.95 (6.41)	<0.0001	15.11 (5.95)	14.50 (4.36)	16.04 (5.64)	0.146
Selenium (mg)	0.07 (0.024)	0.087 (0.022)	0.07 (0.042)	0.003	0.07 (0.038)	0.07 (0.031)	0.08 (0.027)	0.107

Food groups								
Grains (g)	351.3 (112.41)	373.38 (101.06)	413.02 (187.26)	0.017	365.47 (154.53)	370.06 (154.85)	391.72 (118.68)	0.416
Fruits (g)	254.03 (194.50)	296.13 (179.97)	330.29 (245.52)	0.065	328.02 (287.27)	247.52 (130.37)	305.67 (201.96)	0.057
Vegetables (g)	262.34 (137.56)	302.27 (162.98)	379.23 (236.26)	<0.0001	323.03 (179.51)	281.11 (166.06)	331.09 (205.74)	0.183
Meat and its products (g)	114.90 (54.43)	120.79 (49.77)	143.76 (62.21)	0.003	119.27 (59.44)	124.64 (50.65)	130.99 (59.48)	0.428
Beans (g)	28.32 (20.82)	32.04 (21.83)	41.30 (42.75)	0.019	35.44 (43.03)	34.07 (29.92)	32.56 (21.12)	0.837
Dairy products (g)	379.33 (288.56)	335.29 (195.25)	386.30 (266.14)	0.384	317.99 (237.19)	411.13 (278.95)	361 (232.58)	0.100
Nuts and seeds (g)	13.78 (11.73)	16.57 (9.84)	19.23 (18.61)	0.043	15.71 (17.59)	14.69 (1.44)	18.82 (14.32)	0.045
Fats (g)	16.79 (13.54)	20.73 (12.83)	32.62 (15.88)	<0.0001	16.62 (12.26)	17.33 (7.73)	31.03 (17.84)	0.0001

SFA = saturated fatty acids, MUFA = monounsaturated fatty acids, and PUFA = polyunsaturated fatty acids. ^*∗*^The *P* value is reported from covariance analysis, and the results are based on mean ± SD. ^*∗*^All of the variables are adjusted for energy intake. *P*^*∗*^*P* value <0.05 shows a significant level of association.

**Table 9:** Appetite, mood, and anthropometric indices in tertiles of vegetable liquid oil and animal fat.

			Tertiles of edible oils							
Variable			Tertile of vegetable liquid oil (g/d)			*P* value	Tertile of animal fat (g/d)			*P* value
		Participants	*T*1: <6	*T*2: 6–9	*T*3: >9		*T*1: <2.08	*T*2: 2.08–4.48	*T*3: >4.48	
		*N* = 245	*N* = 85	*N* = 81	*N* = 79		*N* = 59	*N* = 78	*N* = 108	
Appetite score	Crude	51.8 (13.2)	52.6 (14)	50.6 (12.2)	52.3 (13.4)	0.579	50.3 (15)	50.8 (10.7)	53.4 (13.8)	0.270
	^1^Model 1	—	51.9 (1.4)	51.2 (1.4)	52.4 (1.5)	0.841	50.5 (1.6)	50.2 (1.4)	53.7 (1.2)	0.133
	^2^Model 2	—	51.9 (1.42)	50.4 (1.44)	53.2 (1.52)	0.427	51.1 (1.67)	50 (1.47)	53.5 (1.25)	0.198

Mood score	Crude	29.5 (32.6)	30.5 (31.3)	22.6 (27.8)	35.5 (37.4)	0.041	33.1 (36.2)	26.7 (28.5)	29.6 (33.5)	0.527
	^3^Model 1	—	32.5 (3.4)	24.8 (3.5)	31.2 (3.6)	0.256	34.2 (4.1)	28 (3.5)	28 (3)	0.427
	^4^Model 2	—	32.9 (3.33)	26.1 (3.35)	29.4 (3.55)	0.361	33.7 (3.9)	29.6 (3.47)	27.1 (2.92)	0.415

Overweight and obesity	Crude	24.8 (3.9)	25.1 (3.9)	24.5 (3.6)	24.6 (4.2)	0.675	24.5 (3.9)	25.1 (4.1)	24.7 (3.7)	0.635
	^5^Model 1	—	25.3 (0.42)	24.3 (0.42)	24.7 (0.45)	0.293	24.5 (0.50)	25.2 (0.44)	24.6 (0.37)	0.503
	^6^Model 2	—	25.1 (0.42)	24.3 (0.43)	24.8 (0.44)	0.458	24.7 (0.50)	25.1 (0.44)	24.5 (0.37)	0.579

Waist circumference	Crude	80 (13)	78.9 (14.4)	80.8 (10.9)	80.4 (13.5)	0.630	78.8 (16.2)	81 (12.8)	79.9 (11.2)	0.611
	^3^Model 1	—	78.2 (1.1)	81 (1.1)	80.8 (1.2)	0.188	79.3 (1.3)	80.4 (1.2)	80 (1)	0.835
	^6^Model 2	—	79.1 (1.47)	79.8 (1.49)	81.1 (1.53)	0.643	79.4 (1.74)	81 (1.52)	79.6 (1.29)	0.745

Waist-to-height ratio	Crude	0.49 (0.07)	0.48 (0.08)	0.49 (0.06)	0.49 (0.08)	0.705	0.48 (0.09)	0.50 (0.07)	0.49 (0.06)	0.473
	^3^Model 1	—	0.49 (0.009)	0.49 (0.009)	0.49 (0.009)	0.879	0.48 (0.010)	0.50 (0.009)	0.49 (0.007)	0.414
	^6^Model 2	—	0.48 (0.009)	0.49 (0.009)	0.49 (0.009)	0.743	0.48 (0.010)	0.49 (0.009)	0.49 (0.008)	0.699

^*∗*^The crude model was resulted from one-way ANOVA, and the numbers are reported as mean ± SD. ^*∗*^Models 1 and 2 was resulted from covariance analysis, and the numbers are reported as mean ± SE. ^*∗*^Model 2 is the main model. *P*^*∗*^*P* value <0.05 shows a significant level of association. ^1^Adjusted for energy, age, COVID-19, and body mass index; ^2^model 1 + micronutrients (thiamine, niacin, vitamin B12, iron, and zinc), supplements (zinc, multivitamin, iron + vitamin D, iron, iron + multivitamin, zinc + vitamin D, and multivitamin + zinc + omega-3), and macronutrient (fiber, simple sugar); ^3^adjusted for energy, age, COVID-19, body mass index, and socioeconomic status; ^4^number 3 + micronutrients (thiamine, riboflavin, niacin, B6, folate, vitamin B12, iron, zinc, and magnesium), supplements (vitamin D, zinc, multivitamin, iron + vitamin D, iron, iron + multivitamin, zinc + vitamin D, multivitamin + calcium-D, and multivitamin + zinc + omega-3), and macronutrient (fiber and simple sugar); ^5^adjusted for energy, age, COVID-19, and socioeconomic status; ^6^number 5 + micronutrients (calcium and caffeine), supplements (vitamin D, zinc, multivitamin, calcium, iron + vitamin D, iron, iron + multivitamin, zinc + vitamin D, multivitamin + calcium-D, multivitamin + zinc + omega-3, and iron + calcium), and macronutrient (fiber and simple sugar).

**Table 10:** Odds ratio and 95% confidence interval for unfavorable variables in tertiles of edible oils.

		Tertiles of edible oils							
Variable		Tertile of vegetable liquid oil (g/d)			*P* trend	Tertile of animal fat (g/d)			*P* trend
		*T*1: <6	*T*2: 6–9	*T*3: >9		*T*1: <2.08	*T*1: 2.08–4.48	*T*3: >4.48	
		*N* = 85	*N* = 81	*N* = 79		*N* = 59	*N* = 78	*N* = 108	
High appetite	Crude	1	1.8 (0.9–3.6)	0.9 (0.5–1.9)	0.975	1	0.9 (0.4–2.1)	0.6 (0.2–1.2)	0.111
	^1^Model 1	1	1.5 (0.7–3.2)	0.8 (0.4–1.7)	0.489	1	1 (0.4–2.4)	0.5 (0.2–1.1)	0.073
	^2^Model 2	1	1.26 (0.5–3.02)	2.50 (1.02–6.11)	0.712	1	2.19 (0.86–5.5)	1.98 (0.8–4.4)	0.141

Poor mood	Crude	1	1.6 (0.8–3.2)	0.4 (0.3–1.2)	0.186	1	0.8 (0.3–1.6)	0.8 (0.4–1.6)	0.681
	^3^Model 1	1	1.6 (0.7–3.3)	0.9 (0.4–1.9)	0.878	1	0.7 (0.3–1.6)	0.9 (0.4–2)	0.959
	^4^Model 2	1	0.89 (0.3–2.4)	1.4 (0.6–3.4)	0.726	1	1.01 (0.4–2.3)	0.5 (0.2–1.7)	0.787

Overweight and obesity	Crude	1	0.9 (0.5–1.7)	1 (0.5–1.9)	0.943	1	0.9 (0.4–1.8)	1 (0.5–1.9)	0.897
	^5^Model 1	1	1.2 (0.6–2.3)	1.1 (0.5–2.2)	0.713	1	0.9 (0.4–1.9)	1 (0.5–2.1)	0.772
	^6^Model 2	1	0.99 (0.4–2.03)	1.03 (0.49–2.1)	0.989	1	0.9 (0.4–1.9)	0.88 (0.4–1.7)	0.820

High waist circumference	Crude	1	0.8 (0.4–1.8)	0.7 (0.3–1.6)	0.532	1	1 (0.4–2.3)	1.2 (0.5–2.6)	0.590
	^3^Model 1	1	0.5 (0.1–1.5)	0.5 (0.1–1.7)	0.297	1	1.2 (0.4–3.8)	1.3 (0.4–3.6)	0.632
	^6^Model 2	1	1.53 (0.6–3.6)	1.47 (0.6–2.7)	0.626	1	0.70 (1.46–4.3)	0.46 (0.3–1.26)	0.280

Waist-to-height ratio	Crude	1	0.9 (0.5–1.7)	0.9 (0.4–1.7)	0.801	1	0.8 (0.4–1.7)	1.1 (0.6–2.2)	0.499
	^3^Model 1	1	1.2 (0.6–2.3)	0.9 (0.4–1.8)	0.927	1	0.8 (0.4–1.7)	1.2 (0.6–2.4)	0.405
	^6^Model 2	1	1.2 (0.5–2.4)	1.29 (0.6–2.7)	0.626	1	0.7 (0.3–1.4)	0.46 (0.3–1.26)	0.280

^*∗*^The *P* trend was reported from logistic regression, and the results are based on the odds ratio or OR (95% CI). ^*∗*^Model 2 is the main model. *P*^*∗*^*P* value <0.05 shows a significant level of association. ^1^Adjusted for energy, age, infected with COVID-19, and body mass index; ^2^model 1 + micronutrients (thiamine, niacin, vitamin B12, iron, and zinc), macronutrient (fiber and simple sugar), supplements (zinc, multivitamin, iron + vitamin D, iron, iron + multivitamin, zinc + vitamin D, and multivitamin + zinc + omega-3), and socioeconomic status; ^3^adjusted for energy, age, infected with COVID-19, body mass index, and socioeconomic status; ^4^number 3 + micronutrients (thiamine, riboflavin, niacin, B6, folate, vitamin B12, iron, zinc, and magnesium), macronutrient (fiber and simple sugar), supplements (vitamin D, zinc, multivitamin, iron + vitamin D, iron, iron + multivitamin, zinc + vitamin D, multivitamin + calcium-D, and multivitamin + zinc + omega-3); ^5^adjusted for energy, age, infected with COVID-19, and socioeconomic status; and ^6^number 5 + micronutrients (calcium and caffeine), supplements (vitamin D, zinc, multivitamin, calcium, iron + vitamin D, iron, iron + multivitamin, zinc + vitamin D, multivitamin + calcium-D, multivitamin + zinc + omega-3, and iron + calcium), and macronutrient (fiber and simple sugar).

## Data Availability

The data sets used and/or analyzed during the current study are available from the corresponding author upon reasonable request.
